# Evaluation of Colonization by *Candida albicans* and Biofilm Formation on 3D-Printed Denture Base Resins

**DOI:** 10.3390/ma18215018

**Published:** 2025-11-04

**Authors:** Pedro Guilherme Lemos Corrêa, Sarah Ribeiro Cruz-Araújo, Carolina Alves Freiria de Oliveira, Raiane Rodrigues da Silva, Viviane de Cássia Oliveira, Valéria Oliveira Pagnano, Claudia Helena Silva-Lovato, Rodrigo Galo, Arunas Stirke, Wanessa C. M. A. Melo, Ana Paula Macedo

**Affiliations:** 1 Deparatamento de Materiais Dentários e Prótese, Faculdade de Odontologia de Ribeirão Preto, Universidade de São Paulo, Ribeirão Preto 14040-904, SP, Brazil; pedroglcorrea@usp.br (P.G.L.C.); sarahrcruz_araujo@usp.br (S.R.C.-A.); carolina.alves.oliveira@usp.br (C.A.F.d.O.); vivianecassia@usp.br (V.d.C.O.); valpag@forp.usp.br (V.O.P.); chl@forp.usp.br (C.H.S.-L.); anamacedo@usp.br (A.P.M.); 2 Department of Functional Materials and Electronics, Center for Physical Sciences and Technology, LT-02300 Vilnius, Lithuania; raiane.r.silva@ufv.br (R.R.d.S.); arunas.stirke@ftmc.lt (A.S.); wanessa.melo@ftmc.lt (W.C.M.A.M.); 3 Centro de Investigación Biomédica en Red de enfermedades respiratorias (Ciberes), Hospital Clinic de Barcelona, University of Barcelona, 08036 Barcelona, Spain; 4 Departamento de Ortopedia e Anestesiologia, Faculdade de Medicina de Ribeirão Preto, Universidade de São Paulo, Avenida Bandeirantes 3900, Ribeirão Preto 14040-904, SP, Brazil

**Keywords:** 3D impression, acrylic resin, biofilm, *Candida albicans*, extracellular polymeric substances, denture bases

## Abstract

Beyond mechanical performance and aesthetics, the susceptibility of 3D-printed resins to microbial colonization and biofilm formation represent an important factor influencing dentures’ longevity. Therefore, this study evaluated *Candida albicans* colonization and mature biofilm formation on three different 3D-printed denture base resins (Bio Denture—BD; Denture Base Cosmos—CD; Smart Print Bio Denture—SP) and compared them to heat-curing resin (HC). Before the microbiological evaluation, the surface roughness (Sa) was assessed. Biofilm viability was determined through colony-forming units per milliliter (CFU/mL) and biofilm morphology was qualitatively examined using a scanning electron microscope (SEM). The composition of the extracellular polymeric substance (EPS) was investigated by measuring the amounts of carbohydrates (µg/mL), proteins (ng/mL), and extracellular DNA (eDNA) (fluorescence unit). One-way ANOVA was performed for eDNA and Sa and Kruskal–Wallis for the other properties (α = 0.05). Higher surface roughness mean values (standard deviation) (*p* < 0.05) were observed in CD [0.111 (0.013)] compared to HC [0.084 (0.018) and BD [0.078 (0.015)]. For wettability, BD [63.2 (5.2)] and SP [65.2 (3.1)] resins showed a greater wettability (*p* < 0.05) than HC resin [73.0 (3.5)], while SP showed lower (*p* < 0.01) protein levels (425 ng/mL) compared to HC (568.6 ng/mL) and BD (554.8 ng/mL) in the EPS. Despite these differences, the 3D-printed denture base resins exhibited microbial load (CFU/mL), EPS composition (carbohydrates and eDNA), and morphological features of *C. albicans* biofilm comparable to those of conventional heat-cured PMMA. These findings suggest that, despite resin-specific variations, 3D-printed denture base materials exhibit a similar susceptibility to *C. albicans* colonization and biofilm formation as conventional denture bases, thereby directing future research towards developing new 3D-printed resins with enhanced antimicrobial properties to improve clinical outcomes.

## 1. Introduction

Complete and partial removable dentures remain a widely used approach for restoring lost oral functions, such as chewing, speaking, swallowing and aesthetics, due to edentulism [1]. Commonly, heat–heat-cured polymethylmethacrylate (PMMA) resin is the gold standard material used in the manufacturing of these devices, owing to characteristics including aesthetic benefits, ease of handling and pigmentation, cost-effectiveness, and low toxicity [2,3].

Although the use of heat–heat-cured PMMA is widely recognized, in the last decade, digital dentistry, represented by computer-aided design and manufacturing (CAD/CAM), has established itself as a significant alternative to the conventional manufacturing process of removable dentures [4,5]. These techniques have become increasingly adopted among dentists, mainly because of their capacity of optimizing the manufacturing workflow by reducing the frequency of appointments and chairside and laboratory time [6]. CAD/CAM technology is composed primarily of two specific methods: subtractive and additive manufacturing.

Subtractive manufacturing [7] involves the controlled removal of material from a pre-fabricated block of resin, until the desired shape is obtained, employing burs and discs attached to a milling machine [8]. Although this method is capable of producing the intended shape, the waste of material is a significant disadvantage, since most of the block is not used in the fabrication of the final piece [5,8]. In this context, additive manufacturing (3D-printing) has emerged as a more recent alternative in digital dentistry, which stands out due to its reduced material waste, higher level of reproducibility and its capacity of printing customized and complex designs [9,10].

Additive manufacturing is a layer-by-layer process in which the final product is built sequentially, with stereolithographic-based techniques being the most widely used approach [11]. Among additive manufacturing strategies, the digital light processing (DLP) strategy employs a digital micromotor device to generate a light mask that rapidly photopolymerizes an entire layer at once [12]. DLP technology has been increasingly used in dentistry. However, few studies have addressed the adhesion of microorganisms on the surface of printed resins, and none have evaluated the extracellular matrix of these biofilms [13].

Denture stomatitis, an inflammatory disease of the mucosa supporting dentures, is often associated with *Candida albicans* and commonly occurs in patients with inadequate oral hygiene or compromised immunity [14]. Biofilm formation on denture bases, including those produced by 3D-printing technologies, represents a significant clinical concern in these populations [15]. It is important to note that biofilms are not homogeneous structures; their architecture and composition can vary significantly across a surface [16]. This heterogeneity contributes to the challenges in treatment, as biofilm-associated pathogens are also linked to systemic and hospital-acquired infections [16]. The study of *C. albicans* is particularly relevant as it is a notorious pathogenic fungus whose robust biofilm formation, conferring significant treatment resistance [17], is a key factor in devastating systemic and hospital-acquired infections [18].

Many studies evaluate the adhesion of microorganisms based solely on colony-forming unit counts (CFU). However, it has already been stated that biofilm formation is influenced by both external and internal factors [16]; therefore, studying the composition of EPS is essential for understanding the physical structure and mechanical strength of the biofilm. The presence of polysaccharides in the biofilm extracellular polymeric substances (EPS) is associated with microbial cell adhesion as it forms the essential skeleton of the biofilm. In addition, proteins contribute to the hydrophobicity of the biofilm, and sessile structures with high protein content are typically more hydrophobic. The presence of extracellular DNA (eDNA) enhances cell-to-cell adhesion and supports the structural integrity of the biofilm.

Despite the growing number of studies on 3D-printed dental resins, little is known about the composition of biofilms formed on these materials. Many studies evaluate the adhesion of microorganisms based just on colony-forming unit counts, without assessing the other components of the biofilm, particularly extracellular polymeric substances (EPS). Therefore, the aim of this study was to investigate the colonization and formation of mature biofilm by *Candida albicans* on the surface of three different 3D-printed resins, using heat-cured acrylic resin as a control. The hypothesis was that the printed resins would not show significant differences in the analysis and would exhibit similar microbial adhesion and biofilm formation, supporting their use in the manufacture of dental prosthesis bases.

## 2. Materials and Methods

### 2.1. Production of Specimens

A total of 52 circle specimens (Ø20 mm × 3 mm) were fabricated, comprising (1) Bio Denture—Prizma^®^ (n = 13) (BD); (2) Denture Base Cosmos—Yller^®^ (n = 13) (CD); (3) Smart Print Bio Denture—Smart Dent^®^ (n = 13) (SP); and (4) conventional PMMA acrylic resin (n = 13) (HC). The specimens of conventional heat-cured acrylic resin (Clássico Artigos Odontológicos Clássico Ltda., São Paulo, Brazil) were produced using dental gypsum type III and IV molds. The molds were made using metal matrices and contained in muffle systems (John Indústria Brasileira, São Paulo, Brazil). The heat-cured resin specimens were polymerized after pressing, as indicated by the manufacturer in an electronic polymerizer (Termocicler 100, Oficina de Precisão da Prefeitura do Campus da USP de Ribeirão Preto, Ribeirão Preto, Brazil).

For manufacturing the 3D-printed resin specimens, digital designs were initially prepared using the Rhinoceros software, version 6.0 (Robert McNeel & Associates, Seattle, WA, USA). The design was then exported in STL (Standard Triangle Language) format to Chitubox Basic 1.9.1 software (CBD Technology Co., Shenzhen, China), where supports were added and the file was sliced into layers for printing. The printing parameters were applied according to those indicated by the respective manufacturers (Table 1) and they were adjusted after printing test patterns that allowed us to verify that the final print corresponded to the expected dimensions of the specimens. The printer used was Mars 4 (Elegoo, Shenzhen, China).

### 2.2. Finishing and Polishing of Specimens

The removal of the excesses of heat–heat-cured acrylic resin and the removal of the supports from the 3D-printed specimens were performed using a fine-cut bur attached to a micromotor. Finishing and polishing were performed mechanically for all experimental groups, equally and for a standardized time, using a horizontal polishing machine (Panambra Industrial e Técnica SA, São Paulo, Brazil). Firstly, a sequence of sandpapers (150, 320, 600, 1200, and 2000 grit, Norton Abrasivos Brasil, Guarulhos, Brazil) was used, followed by a polishing cloth with a 1 μm alumina suspension (Fortel Indústria e Comércio Ltda., São Paulo, Brazil), and then a polishing cloth with a calcium carbonate (Quimidrol Comércio, Indústria e Importação Ltda., Joinvile, Brazil) and water solution.

### 2.3. Analysis of the Surface Roughness

The specimen’s surface roughness (Sa) was assessed using a confocal laser scanning microscope (LEXT OLS4000, Olympus, Tokyo, Japan) to ensure it was aligned with the literature’s recommended standard of 0.2 μm [19]. In this analysis, four regions of each specimen were evaluated, using a 10× magnification lens and a field of view of 2574 µm × 2577 µm. The average roughness was quantified using software (LEXT 3D Measuring Laser Microscope OLS4000 version 3.1.15.1, Olympus, Japão) and reported in micrometers (µm).

### 2.4. Analysis of Surface Wettability

Wettability was evaluated by measuring the right and left contact angles of a sessile drop (5 µL) of artificial saliva on the surface of the samples. Three drops per specimen were evaluated using a goniometer (model OCA20, DataPhysics Instruments GmbH, Filderstadt, Germany). Finally, the average contact angle of each specimen was obtained.

The composition of the artificial saliva is characterized by 4 g/L of hydroxyethylcellulose, 1 g/L of potassium chloride, 1 g/L of sodium chloride, 50 mg/L of magnesium chloride, 400 mg/L of potassium phosphate, and 2 mg/L of methyl 4-hydroxybenzoate (pH = 7) [20].

### 2.5. Biofilm Formation

Firstly, for the disinfection of the specimens, they were left immersed for 30 min in isopropyl alcohol. Afterwards, they were individually placed into the wells of a 6-well cell culture plate (Kasvi, Pinhais, Brazil). The evaluation of microbial adhesion and biofilm formation was carried out in triplicate, both technically and biologically (n = 9).

The −80 °C frozen stock of *C. albicans* (ATCC 90028) was thawed and cultured in Brain Heart Infusion Agar (BHIA). After 24 h of incubation, one colony was transferred to Brain Heart Infusion Broth (BHI) and the inoculum was prepared when the culture achieved the exponential growth phase. Each well received 5 mL of BHI inoculated at a concentration of 10^7^ CFU/mL, and the plates were kept at 37 °C, under agitation (75 rpm), for 90 min to allow initial yeast adhesion. After this period, the specimens were washed three times with sterile PBS to remove non-adherent yeasts. Then, 5 mL of sterile culture medium was added to each well, and the plates were reincubated, in the same conditions, for 48 h to allow biofilm maturation. Half of the culture medium was replaced after 24 h of incubation.

After 48 h of biofilm maturation, the specimens were removed from the plate, washed three times with PBS to remove planktonic cells and transferred to 50 mL test tubes containing 5 mL of PBS. These tubes were then put in an ultrasonic bath (200 W; 40 kHz) for 30 min (Altsonic; Clean 9CA-Alt Equipamentos Ltda., Ribeirão Preto, Brazil) to detach the biofilm from the specimens’ surfaces. Following this, the tubes were vortexed for 30 s to homogenize the biofilm suspension. Subsequently, 1000 µL of this biofilm suspension was collected in three separate aliquots: one for determining the microbial load (CFU/mL), one for quantification of proteins and carbohydrates in the EPS, and the another one for extraction and quantification of eDNA in the EPS.

### 2.6. Determination of the Colony-Forming Units (CFU/mL)

From the previous 1000 µL of biofilm suspension, 10-fold serial dilutions (10^0^ to 10^−7^) were prepared and subsequently seeded in Petri dishes containing a BHIA. Afterwards, the Petri dishes were incubated at 37 °C for 24 h in a microbiological incubator. The amount of colonies was registered and the estimation of the number of CFU/mL was performed by considering the dilution and the volume seeded. The data were presented in Log_10_CFU/mL.

### 2.7. Extraction of Biofilm’s Extracellular Polymeric Substances (EPS)

The biofilm suspension was vortexed and homogenized with 54 µL of 35% (*v*/*v*) formaldehyde in sterile tubes. The suspension was incubated at room temperature (RT) for 1 h. After that, 200 µL of 1 M NaOH was added to each tube and left for 3 h, at RT. Afterwards, the tubes were centrifuged at 13,500 rpm for 1 h at 4 °C. At this stage, the cells and solid debris were precipitated, and the supernatant, containing solubilized EPS, was collected and filtered, to new sterile tubes, through a 0.22 μm sterile filter membrane (Kasvi, Pinhais, Brazil).

The filtered supernatant was concentrated by ethanol (Labsynth, Diadema, Brazil) precipitation. Thus, 1250 µL of cold ethanol (4 °C) was added to 250 µL of the EPS, and this mixture was centrifuged at 13,500 rpm for 30 min and 4 °C. The supernatant was then discarded and the pellet was left to completely dry at 90 °C in a dry water bath, for 15 min. The resulting dry pellet was dissolved in 250 µL of PBS. This process was repeated twice: once for protein analysis and once for carbohydrate analysis, using separate microtubes for each analysis (Kasvi, Pinhais, Brazil).

### 2.8. Quantification of Proteins in the EPS

The quantification of proteins was performed using the Bradford method, which involves the reaction of Coomassie Brilliant Blue G-250 dye, resulting in a color change that can be measured with a spectrophotometer [21].

First, a standard curve was prepared by diluting bovine serum albumin (BSA) (Sigma-Aldrich, St. Louis, MO, USA) on PBS. In a microtube (Kasvi, Pinhais, Brazil), 20 µL of each sample was combined with 1000 µL of the Bradford reagent mixture and incubated for 5 min at RT. This mixture was later transferred to a microplate and the absorbance was then measured in a spectrophotometer at a wavelength of 595 nm and the protein concentrations in the EPS samples was calculated according to the reading of the standard curve.

### 2.9. Quantification of Carbohydrates in the EPS

Carbohydrate quantification was performed using the Anthrone method [22]. To prepare the standard curve, glucose (Sigma-Aldrich) was diluted in PBS.

Two-hundred and fifty microliters of EPS sample was mixed with the Anthrone reagent (Dinâmica Química Contemporânea LTDA, Indaiatuba, Brazil) (2 g/L) in a 1:4 ratio (sample/Anthrone reagent) in a sterile microtube. The mixture was left to cool to RT before being transferred to a boiling water bath for 10 min. The tubes were then cooled again to RT.

The absorbance of the samples and standard curve were measured using a spectrophotometer at a wavelength of 620 nm. The concentration of polysaccharides was calculated according to the reading of the standard curve.

### 2.10. Extraction and Quantification of the eDNA from the EPS

The biofilm samples were centrifuged at 10.098 rpm for 10 min and 4 °C. The resultant supernatant, containing the eDNA, was then transferred to a new sterile microtube.

The eDNA quantification was performed using an intercalating fluorescent agent (SYBR Green). The SYBR Green dye (Cellco Biotec, São Carlos, Brazil) was diluted in a TE 1× buffer, at a 1:200 proportion. Then, 100 µL of each eDNA sample was added into separate wells of a 96-well Black plate (CLS3925-Corning Inc., Corning, NY, USA). After that, 100 µL of the dye solution was added to each well containing the eDNA sample [23].

The mixture was gently mixed and incubated for 5 min protected from light, at RT. The fluorescence values were measured in a fluorometer (λexc: 480 nm; λemi: 520 nm).

### 2.11. Qualitative Analysis of Biofilm Morphology

For the qualitative analysis of biofilm morphology, after biofilm maturation one specimen of each resin was fixed with 3 mL of 2.5% (*v*/*v*) glutaraldehyde (Sigma-Aldrich), dehydrated in a graded ethanol series [30, 50, 60, 70, 80, 90, 95 and 100% (*v*/*v*)] and immersed in 1 mL of Hexamethyldisilazane (Sigma-Aldrich) for 15 min. Afterwards, each specimen was metal-covered and analyzed using a scanning electron microscope (EVO MA10, Carl Zeiss, Jena, Germany) at ×2000 magnification, with a working distance (WD) of 9 mm and an acceleration voltage of 20 kV.

### 2.12. Data Analyses

All data sets were firstly evaluated for adherence to normal distribution and homogeneity of variances. Surface roughness, molhability and eDNA quantification were analyzed using one-way ANOVA. For microbial load, protein and carbohydrate quantification, the comparisons were carried out using the Kruskal–Wallis test. Multiple comparisons were adjusted using Bonferroni’s correction and a significance level of 5% was considered. The analyses were conducted using IBM SPSS Statistics for Windows, Version 21.0 (IBM Corp, Armonk, NY, USA).

## 3. Results

**Figure 1:**
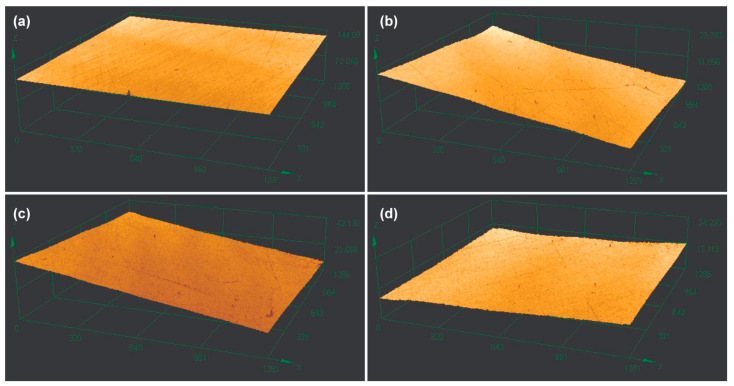
Characteristics of the surface of all resins obtained with laser scanning microscopy. (**a**) HC—heat-cured resin; (**b**) BD—Bio Denture; (**c**) CD—Denture Base Cosmos; (**d**) SP—Smart Print Bio Denture.

The results obtained for surface roughness (Table 2) showed that after finishing and polishing, the specimens presented values lower than maximum recommended in the literature (0.2 μm) [19]. However, a significant difference was found for roughness (*p* = 0.005), with CD showing higher surface roughness values than HC (*p* = 0.037) and BD (*p* = 0.007), even after similar mechanical polishing. Figure 1 shows the images obtained for surface roughness using confocal laser scanning microscopy for all resins.

**Figure 2:**
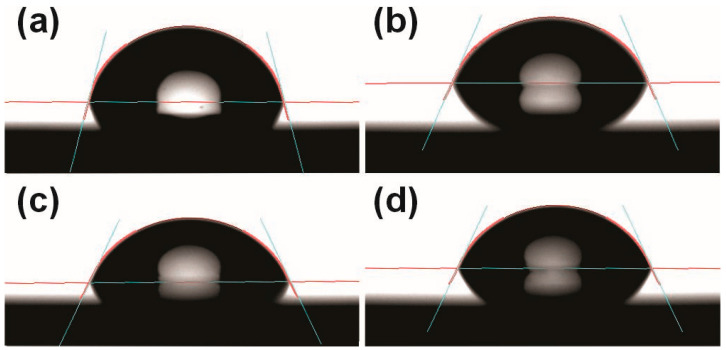
Image of the wettability test obtained using a goniometer. (**a**) HC—heat-cured resin; (**b**) BD—Bio Denture; (**c**) CD—Denture Base Cosmos; (**d**) SP—Smart Print Bio Denture.

For wettability, BD (*p* = 0.004) and SP (*p* = 0.027) resins showed a greater molhability than HC resin, while CD resin showed intermediate values (Table 1). Figure 2 shows the images obtained for the different resins during the wettability evaluation using the OCA20 goniometer.

**Figure 3:**
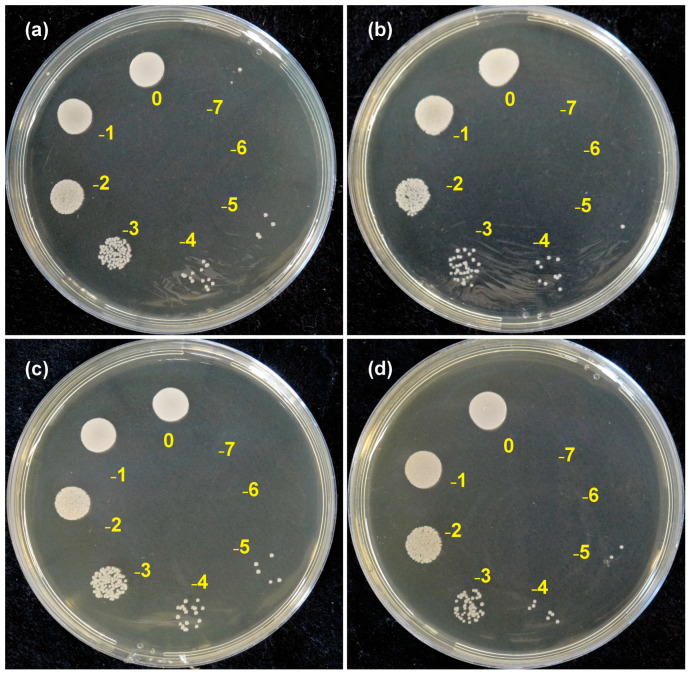
Culture plates containing *C. albicans* growth from serial dilutions of 10^0^ to 10^−7^: (**a**) HC—heat-cured resin; (**b**) BD—Bio Denture; (**c**) CD—Denture Base Cosmos; (**d**) SP—Smart Print Bio Denture.

Figure 3 shows culture plates containing *C. albicans* growth from serial dilutions of 10^0^ to 10^−7^, seeded in BHIA medium after incubation at 37 °C for 24 h to determine the number of CFU/mL.

**Figure 4:**
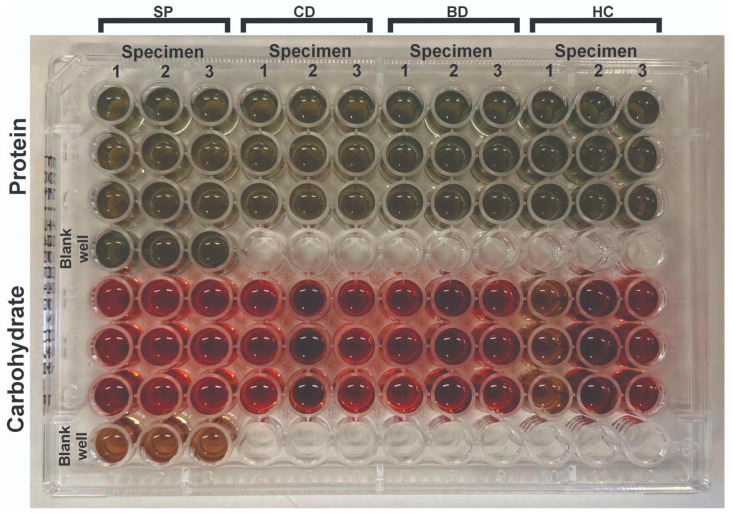
Triplicate quantification of extracellular matrix proteins and carbohydrates: HC—heat-cured resin; BD—Bio Denture; CD—Denture Base Cosmos; SP—Smart Print Bio Denture.

Figure 4 shows a 96-well microtiter plate with the results of biomolecule concentration analyses using the Bradford (for proteins) and Antrona (for carbohydrates) colorimetric methods. The plate contains three specimens per resin analyzed in triplicate.

**Figure 5:**
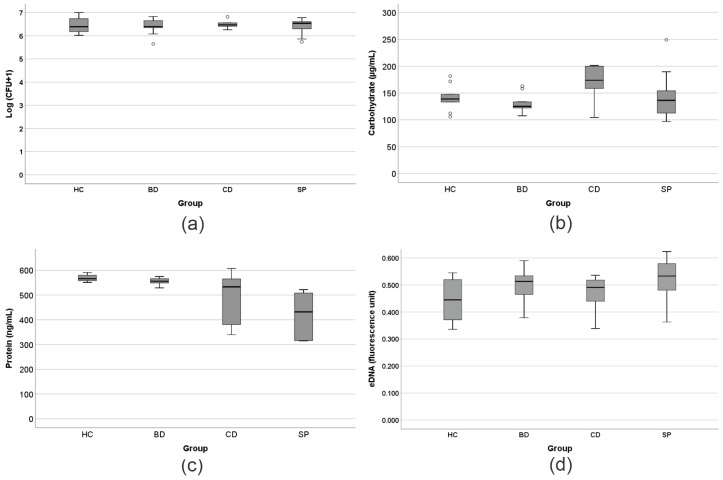
Characteristic of the *C. albicans* biofilm grown on the surface of different 3D-printed resins. (**a**) Microbial load (Log_10_CFU/mL; Kruskal–Wallis test); (**b**) amount of carbohydrate (µg/mL; Kruskal–Wallis test); (**c**) amount of protein (ng/mL; Kruskal–Wallis test); (**d**) quantification of the eDNA (fluorescence units; one-way ANOVA). ⸰ outliers. HC—heat-cured resin; BD—Bio Denture; CD—Denture Base Cosmos; SP—Smart Print Bio Denture.

Figure 5 presents the results obtained for microbial load and the evaluation of the extracellular matrix components of the *C. albicans* biofilms. Overall, there was no significant difference related to the microbial load (*p* = 0.838) and the amount of carbohydrate (*p* = 0.173) and eDNA (*p* = 0.209), regardless of the resin type. However, there was a statistically significant difference between the resins in the amount of protein that makes up the EPS (*p* < 0.001). SP had a lower protein content than BD (*p* = 0.011) and HC (*p* < 0.001), while CD had intermediate values.

**Figure 6:**
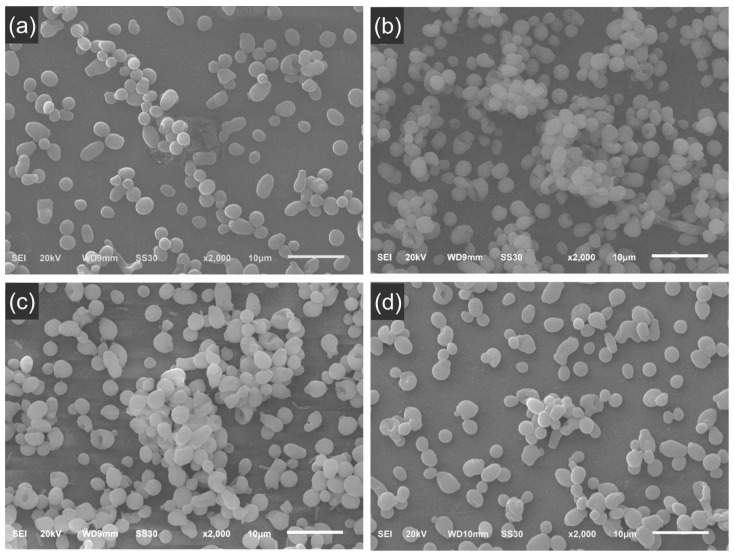
Representative scanning electron micrographs of the *C. albicans* biofilm grown on the surface of different 3D-printed resins. (**a**) HC—heat-cured resin; (**b**) BD—Bio Denture; (**c**) CD—Denture Base Cosmos; (**d**) SP—Smart Print Bio Denture. Magnification 2000×. Scale bar = 10 μm.

The findings regarding the analysis of biofilm morphology (Figure 6) are in agreement with microbial load and characteristics of EPS. Generally, the *C. albicans* biofilm appears similar across the surfaces of all resins evaluated. Slight differences might be observed depending on the selected field of view under SEM examination. No hyphae or pseudohyphae were detected in any of the groups analyzed. The biofilms observed are in an intermediate stage of formation according to Chandra et al. [24].

## 4. Discussion

In this study, despite significant differences in surface roughness, similar microbial load, extracellular carbohydrates, and eDNA were observed for the different resins studied. However, when quantifying the protein in the extracellular matrix, it was observed that the SP printed resin had a lower amount of protein than the HC and BD resins, which leads us to reject the null hypothesis.

*C. albicans* is the primary etiological agent of denture stomatitis, a common oral infection characterized by inflammation of the denture-bearing mucosa, especially in denture wearers with compromised immune systems [25] or inadequate oral hygiene [26]. This microorganism’s ability to colonize denture surfaces has also been established [24]. Once adhered, the fungus grows in a sessile form embedded in an extracellular matrix which covers and protects biofilm cells [17], contributing to the persistence of the infection and increasing its resistance to antifungal treatments [27].

According to our findings, the CD resin exhibited significantly higher surface roughness (Sa) values compared to HC and BD resins. These results are consistent with those reported by Freitas et al. [28], which presented significantly higher values of surface roughness to Cosmos resin (CD), measured by a profilometer (Ra), when compared to two different brands of heat-cured resins. However, this contrasts with the conclusion found in the study of Al-Dwairi et al. [29], which found that the heat-cured resin presented higher surface roughness values (Ra) than 3D-printed resins. However, it is important to mention that the authors investigated resins different from those used in the present study. Since each manufacturer develops its resins with different formulations, the differences may be related to variations in chemical composition, thickness of the printing layers, finishing and polishing method, and material properties.

In this study, some initial disparities arising from the manufacturing processes were likely minimized by standardization in layer thickness (50 µm) and finish, resulting in surfaces equally standardized for microbial adhesion. The determinants of microbial adhesion to resins is considered multifactorial, strongly influenced by surface roughness [13,30], wettability [13], and energy [30]. The absence of significant differences in adhesion between the four resins tested is likely due to the convergence of these essential properties across the different materials. Evidence from previous research challenges the direct correlation between surface roughness and microbial load, highlighting the multifactorial nature of biofilm adhesion [31,32]. The results challenge the assumption that surface roughness solely dictates microbial adhesion [31]. Despite the CD resin exhibiting significantly higher surface roughness (Sa), the similar microbial load on all resins implies that this topographic characteristic was counteracted by other surface properties. Wang et al. [16] describe that factors such as surface chemistry, energy, and wettability likely have a greater influence on the initial attachment of fungal cells than roughness alone. This interaction of factors could explain why the increased roughness of CD did not lead to a higher biofilm load. Furthermore, the roughness surface values for all resins were lower than the maximum value recommended in the literature (0.2 µm), which suggests that there is a statistical difference, but not a clinically relevant one.

The wettability of specimens was evaluated by measuring the contact angle formed between a drop of artificial saliva and the material surface. Lower contact angle values indicate higher wettability and, therefore, a more hydrophilic surface. At the same time, higher values of the contact angles correspond to a more hydrophobic surface, with a lower wettability level [33]. Thus, this property is directly related to microbial adhesion, since more hydrophilic surfaces tend to reduce *C. albicans* adhesion, by decreasing hydrophilic interactions between the microorganism and the surface of the resin [30,34]. On the other hand, more hydrophobic surfaces, associated with higher contact angles, tend to favor fungal adhesion and the beginning of colonization [35,36].

The results of the present study showed greater wettability for 3D-printed resins when compared to heat-cured resin. These findings are consistent with the results reported by Poker et al. [13], which showed higher levels of wettability of the 3D-printed denture base resins when compared to the conventional heat-cured resin, as well as similar *C. albicans* adhesion, since the wettability did not interfere significantly with the adhesion of *C. albicans*. On the other hand, Al-Dwairi et al. [29] found lower contact angle values for the heat-cured resin, when compared to the 3D-printed denture base resins. These differences may be related to the different resins applied in the studies and their different formulations.

Although initial adhesion is determined by surface characteristics, overall mechanical properties (flexural strength, hardness) dictate the long-term clinical integrity of a denture. A material’s resistance to degradation in the oral environment is crucial for maintaining an optimal surface condition against chronic *C. albicans* colonization [37]. The increase in surface roughness tends to facilitate the adhesion of microorganism biofilms not only due to the gain of interaction area between the microorganisms and the material, but also by protecting them from shear forces that could happen in the oral cavity [13,38].

In addition to the similarity in microbial load (CFU/mL), this study demonstrated similar levels of carbohydrates and eDNA in the extracellular matrix of *C. albicans* biofilm. Furthermore, biofilms grown on the surface of different resins showed structural similarity when observed by SEM. The evaluation of biofilm structure by SEM has limitations in terms of visualization of the extracellular matrix, as the serial alcohol dehydration process compromises the integrity of the extracellular matrix, since part of the matrix components are soluble in alcohol [24]. Within the scope of what can be evaluated, the results suggest that, despite the different characteristics of the 3D-printed resins, *C. albicans* showed similar colonization among them, comparable to that observed in the heat-cured resin.

EPS is composed of a range of high-molecular-weight mixtures of polysaccharides, proteins, nucleic acid and inorganic substances that are produced and secreted by the microorganisms [16,39]. These biomolecules provide structural and protective functions in biofilms [40]. Therefore, quantifying the amount of carbohydrates, proteins and eDNA in the extracellular matrix of *C. albicans* biofilm could contribute to understanding the mechanisms behind biofilm growth and strategies to prevent formation on the surface of denture bases.

In the present study, when evaluating the amount of protein in the extracellular matrix, a lower amount of protein was found for SP resin and intermediate values for CD resin. These results can probably be attributed to the influence of stabilizers in their chemical formulation [41], suggesting the formation of a structurally more fragile biofilm that, although not immediately affecting the microbial load, could contribute to a decrease in the fungal load over a longer clinical period. It is important to consider that these two resins contain stabilizers in their formulation. Previous research has suggested that the use of stabilizers in polymers may result in a decrease in the protein load of biofilms [42]. This issue was also explored by Costa et al. [43], who investigated the antimicrobial response of stabilizers used in polymers and found that, although different polymers have antimicrobial activity, their mechanism of action remains poorly understood.

Based on these findings, the lower amounts of proteins observed in the EPS of *C. albicans* biofilms in the SP and CD groups might suggest a more fragile biofilm, contributing to a reduction in fungal load in this resin over longer periods than those evaluated in this study. In addition, lower protein levels might contribute not only to reduced adhesion but also to decreased fungal viability. This is in line with the results found by Abreu-Pereira et al. [44], which reinforced a strong relationship between the composition of the extracellular matrix and biofilm viability. The authors observed that photodynamic therapy against *C. albicans* biofilms promoted significant alterations in the amount of polysaccharides, proteins, and eDNA. The reduction in these biomolecules led to a marked decrease in the fungal burden, as evidenced by lower CFU counts. Although the authors highlight the crucial role of EPS in maintaining biofilm integrity and protecting fungal cells, no effect on microbial load was observed here despite the reduction in proteins.

One limitation of this study is the absence of real-life denture use conditions, which could more accurately reflect the behavior of *C. albicans* biofilm under real circumstances for each type of resin. Another methodological consideration is biofilm thickness, a factor that can interact with surface properties. A uniform thickness across all samples would reinforce the conclusion that surface properties were similar, while significant variations could indicate specific effects of the underlying material not captured by adhesion assays alone [45]. Additionally, a multispecies biofilm analysis would be more representative, considering the microbial diversity of the oral cavity. Therefore, further studies are needed to confirm these results under real-world conditions, incorporating multispecies biofilms, and long-term clinical evaluation. Additionally, investigating the influence of hygiene practices on biofilm development across different resin types may provide critical insights for preventive strategies to reduce microbial colonization and maintain oral health. Overall, the promising results observed for 3D-printed resins encourage continued research into their clinical performance and their role in promoting better oral health outcomes for denture wearers.

## 5. Conclusions

This study demonstrated that *C. albicans* biofilms grown on resins for 3D-printed denture bases (Bio Denture, Denture Base Cosmos, and Smart Print Bio Denture) exhibited comparable levels of microbial load (CFU/mL), biomolecules (carbohydrates and eDNA) from EPS, and similar morphological characteristics when compared to those grown on conventional heat-cured PMMA, despite the greater surface roughness observed in the Denture Base Cosmos resin and lower wettability observed in heat-cured resin. In addition, protein quantification in EPS was lower in Smart Print Bio Denture resin and slightly reduced in Denture Base Cosmos resin, despite its greater roughness. These findings suggest a tendency toward reduced fungal adhesion and viability in these resins, reinforcing their potential advantages for the manufacture of denture bases. Lower biofilm formation contributes to a reduced risk of prosthetic stomatitis and other biofilm-related oral infections.

## Figures and Tables

**Table 1:** Parameters used for printing the specimens.

Resin	Composition	Layer Height (mm)	Exposure Time (s)	Number of Base Layers	Base Exposure Time (s)	Lift/Retract Speed (mm/min)	Post-Curing (Cycles/min)
BD	Proprietary Acrylate Monomers; Pigmentation and Fillers; Acrylate Oligomers; Diphenyl (2,4,6-trimethylbenzoyl) phosphine oxide (photoinitiator)	0.05	3.80	8	35.00	60.0	3/1
CD	Oligomers; Monomers; Photoinitiators; Stabilizer; Pigment	0.05	3.50	10	40.00	65.00	1/10
SP	Monomers, Oligomers, Photoinitiators, Pigments, Stabilizers	0.05	4.30	10	25.00	60.00	1/9

BD—Bio Denture; CD—Denture Base Cosmos; SP—Smart Print Bio Denture.

**Table 2:** Mean (standard deviation) surface roughness (Sa—µm) and wettability (degrees) across the different 3D-printed resin specimens.

Group	Sa	*p*	Wettability	*p*
HC	0.084 (0.018) *	0.005	73.0 (3.5)	0.004
BD	0.078 (0.015) *		63.2 (5.2) **	
CD	0.111 (0.013)		68.7 (2.5)	
SP	0.096 (0.028)		65.2 (3.1) **	

* Significant differences in comparison with CD, ** significant differences in comparison with HC (one-way ANOVA; *p* < 0.05). HC—heat-cured resin; BD—Bio Denture; CD—Denture Base Cosmos; SP—Smart Print Bio Denture.

## Data Availability

The original contributions presented in this study are included in the article. Further inquiries can be directed to the corresponding author.

## Abbreviations

PMMA	Polymethylmethacrylate
CAD/CAM	Computer-aided design and manufacturing
DLP	Digital light processing
CFU	Colony-forming units
EPS	Extracellular polymeric substance
eDNA	Extracellular DNA
SEM	Scanning electron microscopy
Sa	Surface roughness
BD	Bio Denture—Prizma^®^
CD	Denture Base Cosmos—Yller^®^
SP	Smart Print Bio Denture—Smart Dent^®^
HC	Heat-cured polymethylmethacrylate resin
STL	Standard triangle language
rpm	Revolutions per minute
BHIA	Brain heart infusion agar
BHI	Brain heart infusion
PBS	Phosphate-buffered saline
RT	Room temperature
